# Supplementary material for: Immunoglobulin A as a Key Immunological Molecular Signature of Post-COVID-19 Conditions
Source: Viruses. 2023 Jul 13;15(7):1545. doi: 10.3390/v15071545 (PMC10385093; doi:10.3390/v15071545)
Supplement: Supplementary file 1 [file viruses-15-01545-s001.zip › viruses-2469244-supplementary.pdf]

Supplementary Materials

Table S1. Baseline characteristic of the study population.

Variables	Control without PCC (n=34)	Total PCC (n=151)	Subacute (5-12 weeks; n=26)	Chronic (>12 weeks; n=116)
			**Mean (SD)**	
**Age (years)**	35.72 (7.72)	51.82 (14.39)	49.81 (16.90)	52.19 (13.86)
Women	33.41 (7.26)	50.94 (14.23)	50.10 (17.28)	51.28 (13.58)
Men	38.33 (7.60)	54.10 (14.71)	48.83 (17.07)	54.48 (14.49)
**OD (nm)**				
IgM	0.131 (0.088)	0.148 (0.103)	0.124 (0.063)	0.147 (0.105)
IgA	0.375 (0.214)	0.613 (0.349)	0.635 (0.341)*	0.605 (0.352)**
IgG	1.946 (0.269)	1.968 (0.403)	2.067 (0.414)	1.966 (0.397)
			**Nº/Total Nº (%)**	
**Sex**				
Women	18/34 (53%)	109/151 (72%)	20/26 (77%)	83/116 (72%)
Men	16/34 (47%)	42/151 (28%)	6/26 (23%)	33/116 (28%)
**Comorbidities**				
Cardiovascular	-	89/151 (59%)	18/26 (69%)	65/116 (56%)
Metabolic	-	50/151 (33%)	8/26 (31%)	39/116 (34%)
Neuropsychiatric	-	26/151 (17%)	4/26 (15%)	22/116 (19%)
Inflammatory/Systemic	-	26/151 (17%)	6/26 (23%)	18/116 (16%)
Others	-	19/151 (13%)	5/26 (19%)	14/116 (12%)
**Symptons**				
Fatigue	-	120/151 (79%)	19/26 (73%)	94/116 (81%)
Myalgia	-	120/151 (79%)	20/26 (77%)	93/116 (80%)
Memory difficulty	-	113/151 (75%)	17/26 (65%)	89/116 (77%)
Hypoprosexia	-	64/151 (68%)	15/26 (58%)	77/116 (66%)
Headache	-	108/151 (72%)	18/26 (69%)	83/116 (72%)
Anxiety	-	102/151 (68%)	17/26 (65%)	82/116 (71%)
Ageusia	-	97/151 (64%)	16/26 (62%)	73/116 (63%)
Anosmia	-	95/151 (63%)	13/26 (50%)	74/116 (64%)
Reasoning difficulties	-	90/151 (60%)	11/26 (42%)	74/116 (64%)
Paresis	-	78/151 (52%)	15/26 (58%)	62/116 (53%)
Paresthesia	-	71/151 (47%)	9/26 (35%)	56/116 (48%)
Personality change	-	67/151 (44%)	9/26 (35%)	56/116 (48%)
Weight change	-	67/151 (44%)	11/26 (42%)	56/116 (48%)
Sleep disorder	-	67/151 (44%)	8/26 (31%)	59/116 (51%)
Alopecia	-	63/151 (42%)	8/26 (31%)	53/116 (46%)
Depression	-	61/151 (40%)	9/26 (35%)	49/116 (42%)
Dyspnoea	-	60/151 (40%)	14/26 (54%)	46/116 (40%)

Vertigo	-	60/151 (40%)	12/26 (46%)	47/116 (41%)
Arrhythmia	-	53/151 (35%)	11/26 (42%)	42/116 (36%)
Anorexia	-	48/151 (32%)	7/26 (27%)	39/116 (34%)
Aphasia	-	37/151 (25%)	6/26 (23%)	31/116 (27%)
Dyslexia	-	35/151 (23%)	7/26 (27%)	27/116 (23%)
Angina	-	34/151 (23%)	5/26 (19%)	28/116 (24%)
Myoclonus	-	33/151 (22%)	8/26 (31%)	24/116 (21%)
Delirium	-	29/151 (19%)	3/26 (12%)	25/116 (22%)
Hoarseness	-	27/151 (18%)	7/26 (27%)	20/116 (17%)
Dysphagia	-	17/151 (11%)	4/26 (15%)	13/116 (11%)
Thrombosis	-	13/151 (9%)	1/26 (4%)	11/116 (9%)
Bacterial or viral infection	-	9/151 (6%)	3/26 (12%)	5/116 (4%)
Convulsion	-	3/151 (2%)	2/26 (8%)	1/116 (1%)

Statistical analysis was performed using the Kruskall-Wallis test followed by Dunn's post hoc test. The symbols indicate statistical significance in a comparison of immunoglobulin production between patients with or without PCC (* $p < 0.05$; ** $p < 0.01$).

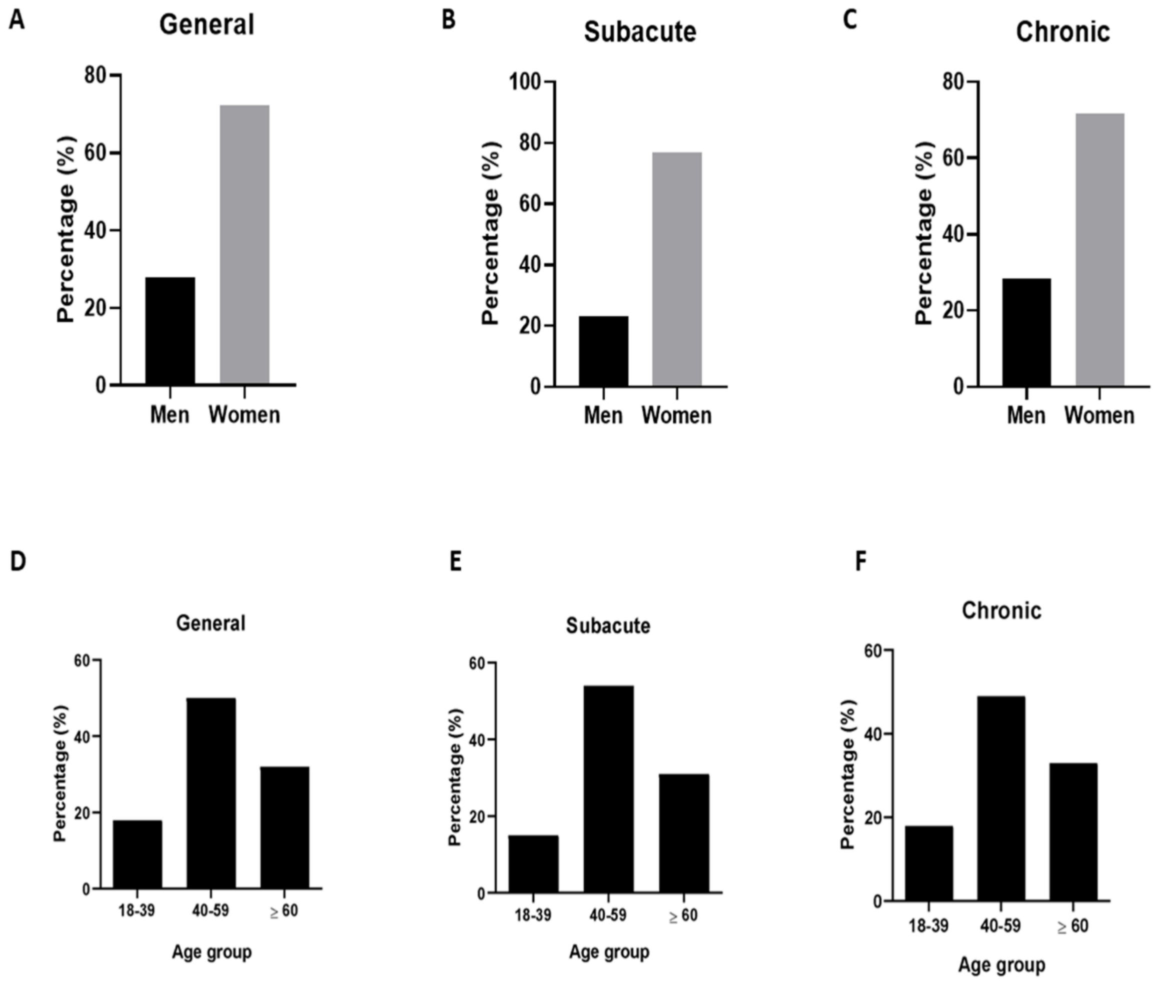

Figure S1. General data of the patients of the Centro de Acolhimento e Reabilitação Pós-COVID-19 (CARP). Percentage of men and women who sought medical assistance at CARP (**A**), divided into subacute (**B**) and chronic (**C**) phases. Percentage of age groups that sought medical assistance at CARP (**D**), divided into subacute (**E**) and chronic (**F**) phases.

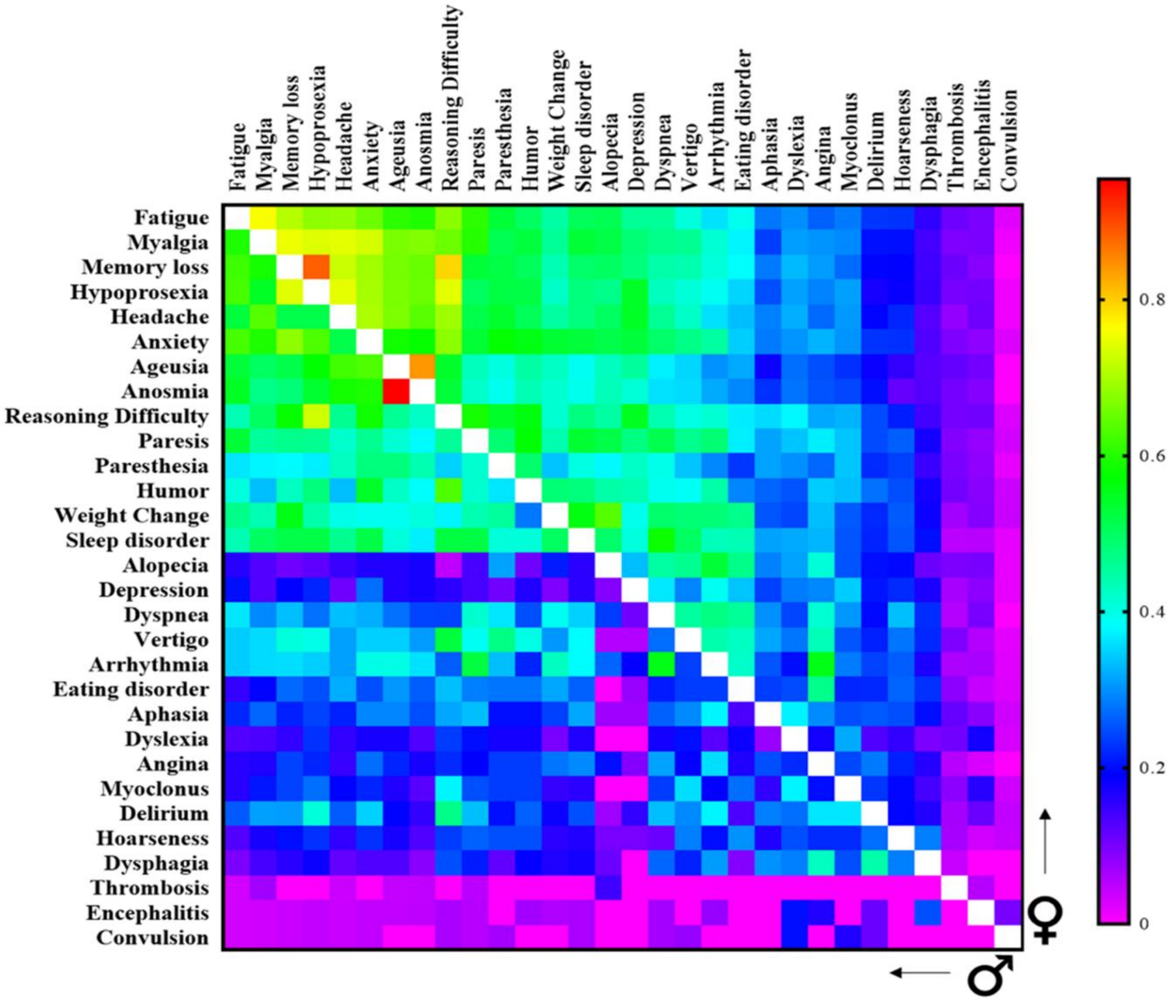

Figure S2. Heatmap showing co-occurrence levels (Jaccard similarity index) between pairs of post-COVID-19 condition symptoms in men and women. The Jaccard index was measured based on the presence/absence of symptoms for each patient. Men are represented by the symbol (♂) and women by the symbol (♀). Increasing similarities are indicated by a warm color. Values equal to 1 are indicated by a blank square.

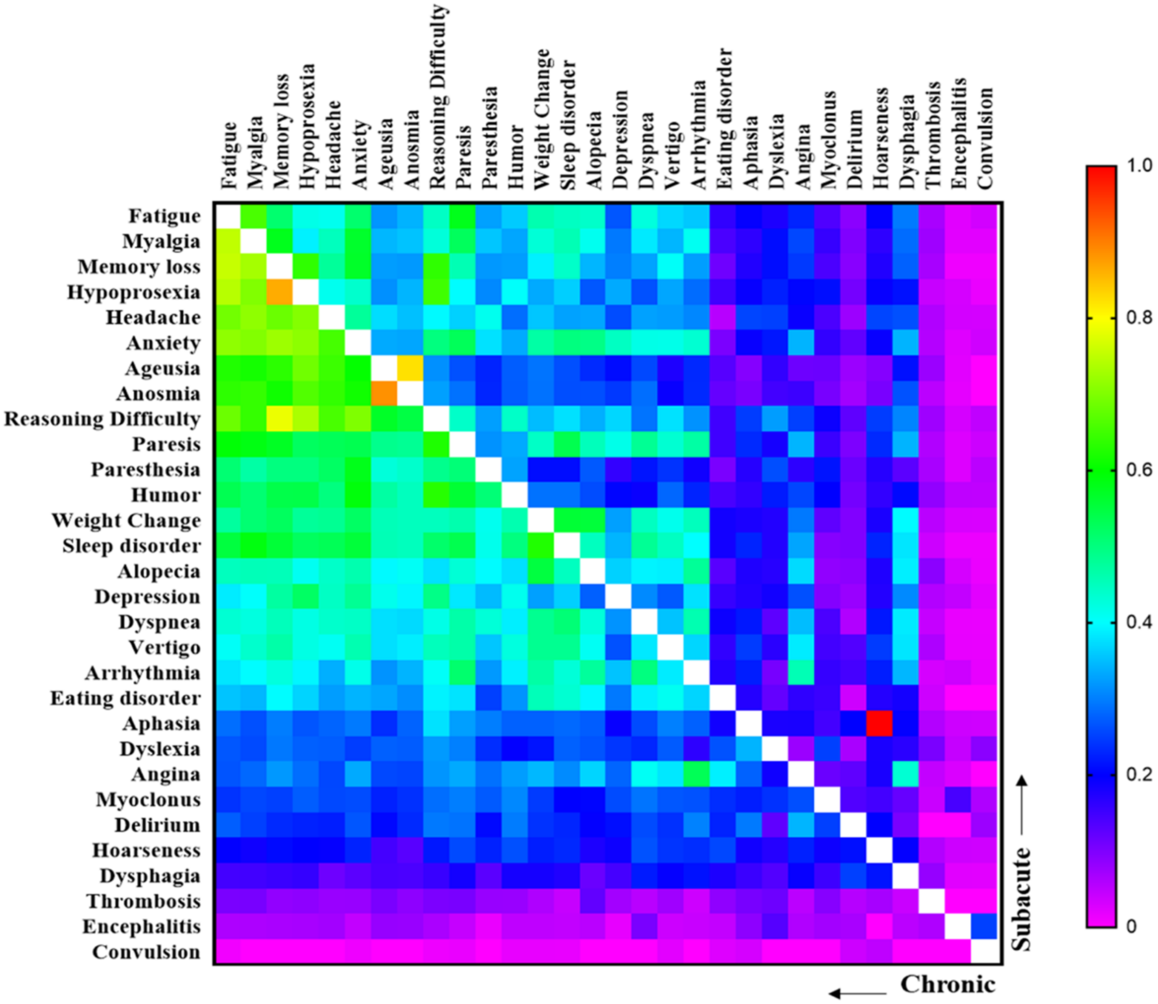

Figure S3. Heatmap showing co-occurrence levels (Jaccard similarity index) between pairs of post-COVID-19 condition symptoms in the subacute and chronic phases of PCC. The Jaccard index was measured based on the presence/absence of symptoms based on the phase of the post-COVID-19 conditions. The co-occurrence of subacute phase symptoms is shown in the upper matrix, while the co-occurrence of chronic phase symptoms is shown in the lower matrix. Increasing similarities are indicated by a warm color. Values equal to 1 are indicated by a blank square.

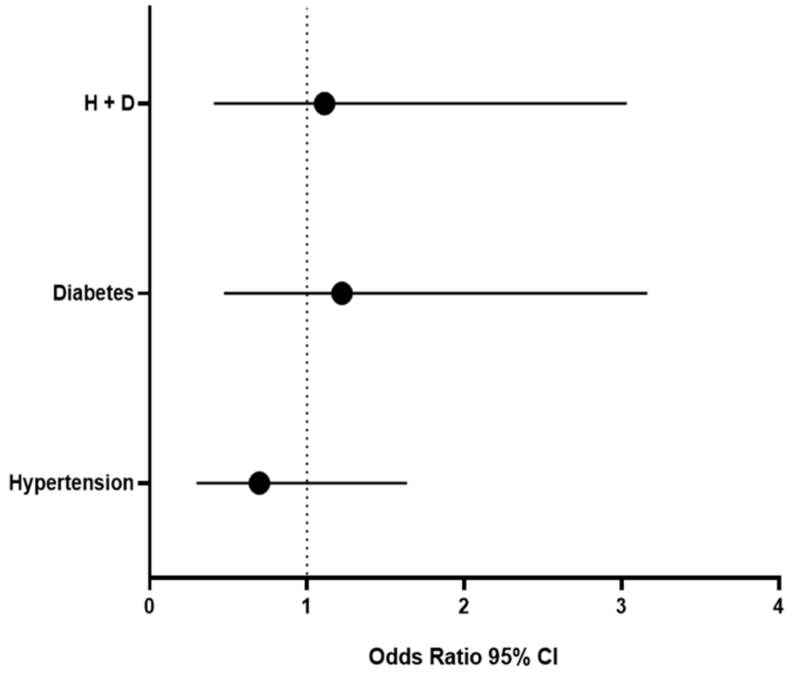

Figure S4. Comorbidities diabetes and hypertension as predisposing factors for the development of post-COVID-19 conditions (PCC). Odds ratio with 95% confidence interval (CI) analysis of comorbidity domains for the development of chronic PCC symptoms. H+D represents patients with diabetes and hypertension comorbidities.

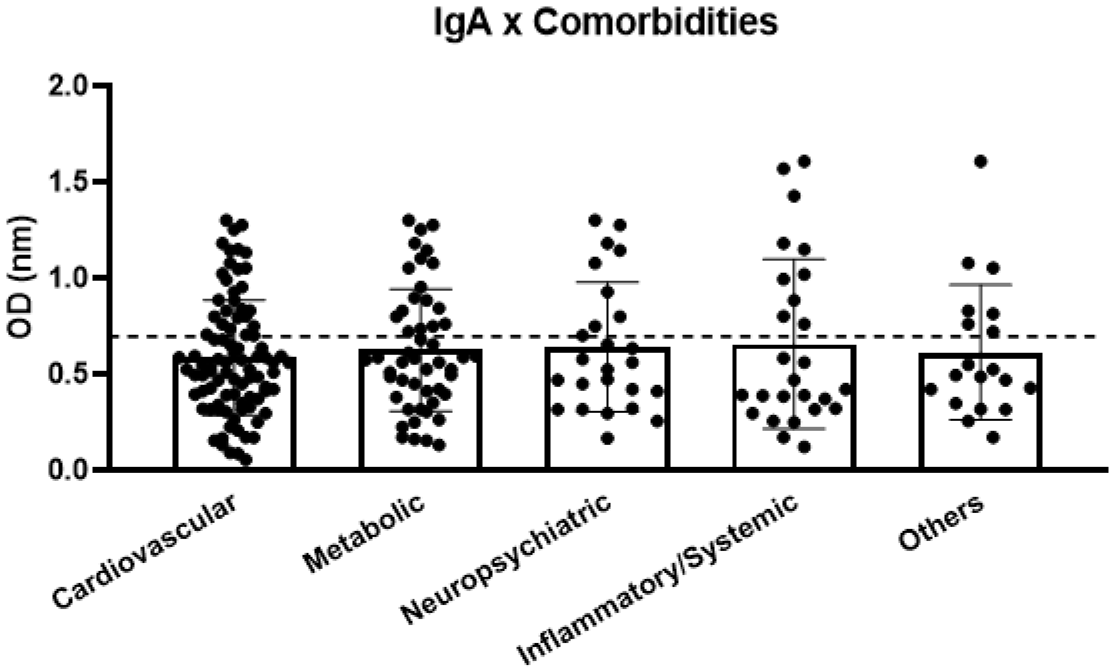

Figure S5. Analysis of correlation between IgA levels and comorbidities domains from 151 PCC patients. Significant statistical differences were performed using the Kruskall-Wallis test followed by Dunn's post hoc test. No statistical significance difference was observed ($p < 0.05$).

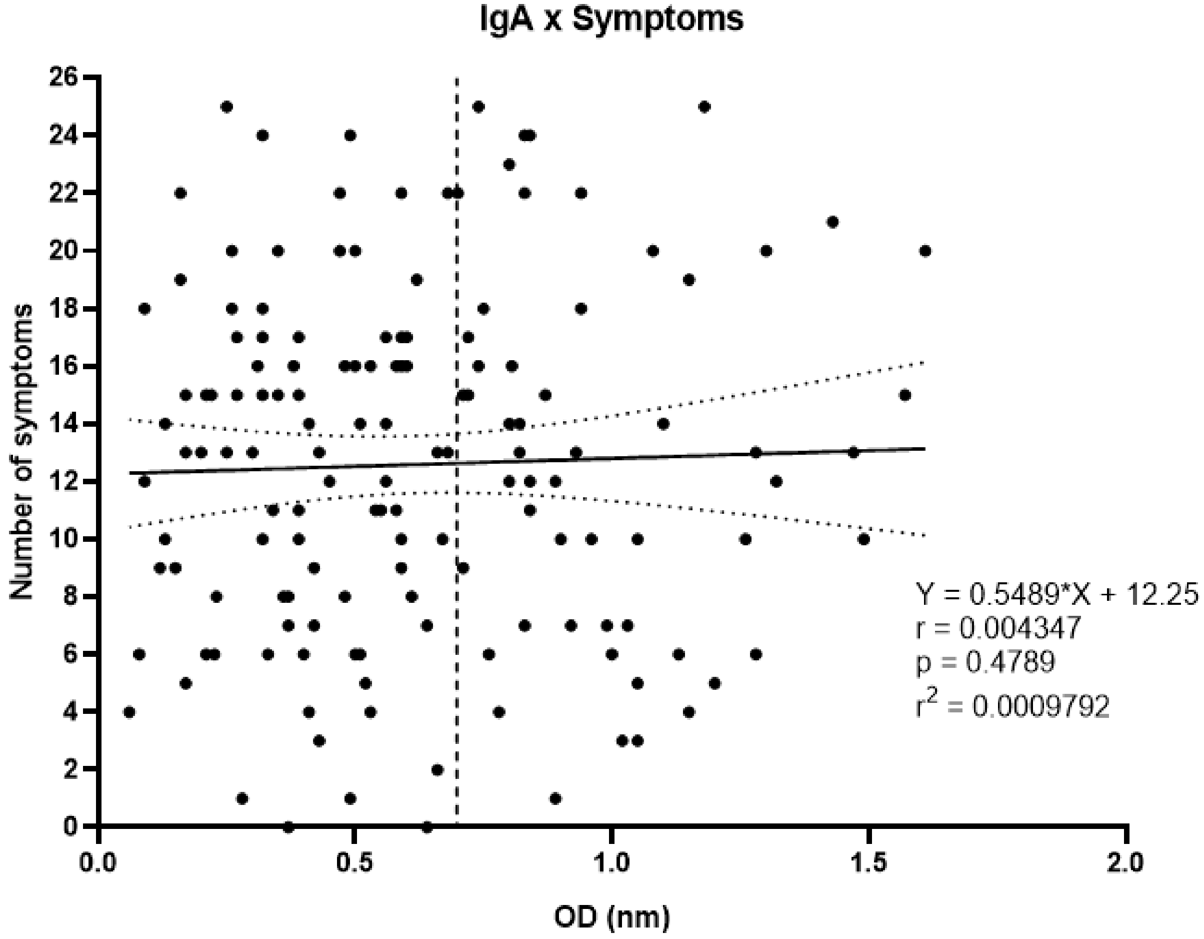

Figure S6. Analysis of the correlation between IgA levels and number of symptoms in 151 PCC patients. Linear Regression of the scatter plot of the number of symptoms present in CARP patients and the OD (nm) values of IgA levels. Values of r, r squared, p and equation indicate that there is no correlation between IgA and number of symptoms. The dashed line (X axis) indicates the cut-off value for IgA (0.698 nm) and the two dotted lines (Y axis) indicate the 95% confidence interval of the straight line.
